# MicroRNA-27a Contributes to Rhabdomyosarcoma Cell Proliferation by Suppressing RARA and RXRA

**DOI:** 10.1371/journal.pone.0125171

**Published:** 2015-04-27

**Authors:** Lucia Tombolan, Matteo Zampini, Silvia Casara, Elena Boldrin, Angelica Zin, Gianni Bisogno, Angelo Rosolen, Cristiano De Pittà, Gerolamo Lanfranchi

**Affiliations:** 1 Department of Biology, University of Padova, Padova, Italy; 2 C.R.I.B.I.-Biotechnology Centre, University of Padova, Padova, Italy; 3 Institute of Pediatric Research, IRP, Padova, Italy; 4 Department of Women’s and Children’s Health, Oncohematology Unit, University of Padova, Padova, Italy; Sun Yat-sen University Medical School, CHINA

## Abstract

**Background:**

Rhabdomyosarcomas (RMS) are rare but very aggressive childhood tumors that arise as a consequence of a regulatory disruption in the growth and differentiation pathways of myogenic precursor cells. According to morphological criteria, there are two major RMS subtypes: embryonal RMS (ERMS) and alveolar RMS (ARMS) with the latter showing greater aggressiveness and metastatic potential with respect to the former. Efforts to unravel the complex molecular mechanisms underlying RMS pathogenesis and progression have revealed that microRNAs (miRNAs) play a key role in tumorigenesis.

**Methodology/Principal Findings:**

The expression profiles of 8 different RMS cell lines were analyzed to investigate the involvement of miRNAs in RMS. The miRNA population from each cell line was compared to a reference sample consisting of a balanced pool of total RNA extracted from those 8 cell lines. Sixteen miRNAs whose expression discriminates between translocation-positive ARMS and negative RMS were identified. Attention was focused on the role of miR-27a that is up-regulated in the more aggressive RMS cell lines (translocation-positive ARMS) in which it probably acts as an oncogene. MiR-27a overexpressing cells showed a significant increase in their proliferation rate that was paralleled by a decrease in the number of cells in the G1 phase of the cell cycle. It was possible to demonstrate that miR-27a is implicated in cell cycle control by targeting the retinoic acid alpha receptor (RARA) and retinoic X receptor alpha (RXRA).

**Conclusions:**

Study results have demonstrated that miRNA expression signature profiling can be used to classify different RMS subtypes and suggest that miR-27a may have a therapeutic potential in RMS by modulating the expression of retinoic acid receptors.

## Introduction

The most common soft tissue childhood sarcoma, rhabdomyosarcoma (RMS) is characterized by two major histological subtypes: alveolar RMS (ARMS) and embryonal RMS (ERMS). The former are linked to a significantly worse prognosis than the latter [1], but the reasons explaining its greater aggressiveness and metastatic potential are largely unknown. ARMS is characterized by the chromosomal translocation t(2;13)(q35;q14) or, less commonly, by the variant translocation t(1;13)(p36;q14), involving *PAX3/FOXO1* or *PAX7/FOXO1* genes, respectively [2]. Several transcriptomic studies have demonstrated that the *PAX/FOXO1* fusion gene confers a particular expression signature to ARMS that clearly distinguishes translocation-positive ARMS from translocation-negative RMS cell lines [3,4]. These findings suggest that despite having a similar morphological appearance, the two subtypes are different biological and clinical entities.

MicroRNAs (miRNAs) are a class of small non-coding RNAs (~22 nucleotides (nt) in length) that negatively regulate protein-coding gene expression post-transcriptionally by targeting mRNAs, mostly at the 3′ untranslated region (3′-UTR) and triggering either translational repression or RNA degradation [5]. It has been estimated that about 30% of the genes are regulated by at least one miRNA which is implicated in a variety of important biological processes including differentiation [6], apoptosis [7], fat metabolism [8], viral infection [9] and tumorigenesis [10,11,12,13]. Since a single miRNA can pleiotropically influence the expression of multiple genes, miRNAs could have a diagnostic value, as showed for tumor classification [14,15]. Several studies have reported that miRNA signatures discriminate different sarcomas including RMS [15,16] and constitute a promising technological advance in molecular techniques which may be able to overcome the difficulties in diagnosing sarcomas [17]. MiRNA expression profiling is, moreover, useful in identifying miRNAs involved in regulating the tumor and constitutes the starting point for functional analysis. Some specific microRNAs, called “metastamiRs,” have recently been demonstrated to play a crucial role not only in controlling the growth of the primary tumor by regulating pathways involved in cell cycle and proliferation, but also in modulating tumor cell migration, invasion, and the interaction with the microenvironment, mechanisms involved in the acquisition of a more aggressive phenotype and promoting the onset of the metastatic process [18,19].

A group of miRNAs, acting as key controllers of skeletal muscle cell fate, have recently been shown to be de-regulated in RMS. The expressions of miR-1 and miR-133a are strikingly decreased in alveolar and embryonal RMS cell lines with respect to differentiated myoblasts and skeletal muscle tissues [20]. MiR-206 in embryonal cell lines also blocks their tumorigenic potential by targeting c-Met mRNA [21] and by directly regulating the *PAX3* gene [16]. Once again, miR-26a promotes myogenesis by targeting the histone methyltransferase (Ezh2) mRNA in RMS cell lines [22]. Since RMS cells fail to complete the skeletal muscle cell differentiation program and irreversibly exit the cell cycle, many studies were focused on a strategy to restore a normal differentiation program. As retinoic acid (RA) has been shown to induce myogenesis in various muscle cells, understanding its role in RMS may help to elucidate mechanisms that can restore proper myogenesis in this type of tumor [23]. Retinoids, also known as ATRA, induce growth inhibition and differentiation of many cell types and are promising agents for the prevention and treatment of several human cancers [24]. Largely utilized for the treatment of acute promyelocytic leukemia, they are under study in other tumors such as breast cancer and neuroblastoma [25,26,27,28]. Several studies have recently demonstrated that RA influences cell proliferation and muscle gene expression in human RMS cell lines [23,29].

MiRNAs have been extensively studied in the context of muscle differentiation, and the miRNA expression patterns identified in RMS have revealed that they are directly involved in tumorigenesis processes. As has already been demonstrated with regard to protein-coding genes, miRNAs can act as oncogenes or tumor suppressors depending on the cellular context in which they are expressed.

Recent studies showing that miR-29 is significantly down-regulated in RMS with respect to what is found in normal skeletal muscle suggest that it may have a tumor suppressor role in RMS [16]. In contrast, miR-183 has been found to be up-regulated in RMS, suggesting that it may play an oncogenic role by targeting the transcription factor EGR1 that regulates the activation of the tumor suppressor gene PTEN [30]. The miR-23a~27a~24–2 cluster has been found to have an altered expression in many diseases, and expression of that cluster has been found to be up-regulated in many cancer types including acute lymphoblastic leukemia [31], chronic lymphocytic leukemia [32], breast cancer [33] and gastric cancer [34,35]. The oncogenic role of miR-27a in tumors has been strongly supported by previous studies. In gastric cancer cells, miR-27a promotes tumor development by targeting the tumor suppressor prohibitin, an evolutionary conserved and ubiquitous protein interacting with pRb and its family members [34]. MiR-27a also contributes to oncogenesis by regulating cell cycle progression in breast cancer cell lines [36]. A recent work conducted in patients with metastatic and recurrent gastric cancer reported that high miR-27a expression is correlated with a significantly worse overall survival. The authors hypothesized that miR-27a could be a useful biomarker predicting a patient’s response to chemotherapy [37]. Taken together, these studies indicate that miR-27a plays a key role in cancer not only in view of its involvement in cell cycle proliferation but also of its utility as a biological marker to refine the prognostic assessment of patients undergoing chemotherapy.

The present study found that translocation-positive ARMS and translocation-negative RMS cell lines are characterized by different miRNA expression signatures that could be used to stratify RMS patients. Attention was focused on the role of miR-27a which is up-regulated in more aggressive RMS cell lines in which it probably acts as an oncogene. We demonstrated that miR-27a is implicated in cell cycle control by targeting the retinoic acid alpha receptor (RARA) and retinoid X receptor alpha (RXRA), suggesting a potential role of miR-27a in drug therapy of rhabdomyosarcoma.

## Material and Methods

### Cell Culture

Human ARMS cells (RH4, RH28, RH30, and RH18), human ERMS cells (RD, RH36, CCA and SMS-CTR) mouse C2C12 myoblast cells and 293T cells were maintained in Dulbecco’s modified Eagle’s medium containing 10% fetal calf serum, penicillin (100 U/mL), and streptomycin (100 ug/mL) (Life Technologies, Carlsbad, CA) at 37°C in 5% CO_2_ in a humidified incubator. RH30, RD and C2C12 cells were obtained from American Type Culture Collection (Manassas, VA); RH18 and RH28 were a gift from Dr. Peter J. Houghton (St. Jude Children’s Hospital, Memphis, TN) [38] RH4 and CCA were gift from Prof. Pier Luigi Lollini (Dept. Medicina Specialistica, Diagnostica e Sperimentale, University of Bologna, Italy) [39,40]. SMS-CTR and RH36 were obtained from Dr. Maria Tsokos (National Cancer Institute, Bethesda, MD) [41].

### Transient transfections of pre-miRNAs or anti-miRNAs

Pre-miRNA negative control (ID 4464058), Anti-miRNA negative control (ID 4464077), miRNA precursors and inhibitors hsa-miR-27a (MIMAT0000084) and hsa-miR-23a (MIMAT0000078) were provided by Ambion (Ambion-Life Technologies, Carlsbad, CA). To obtain transient pre-miR expression or anti-miR expression, RH36 and RH4 cells were respectively plated in 6-well plates at 60–70% confluency and transfected with 50 nM pre-miR-27a/23a/control or with 100 nM anti-miR-27a/23a/control using Lipofectamine 2000 transfection reagent (Life Technologies) following the manufacturer’s instructions. Cells were tested for miR overexpression or downregulation 24h after transfection by qRT-PCR.

### Total RNA and miRNAs isolation

Total RNA was isolated from RMS cell lines using Trizol Reagent (Life Technologies) following the manufacturer’s protocol. The commercially available PureLink miRNA Isolation Kit (Life Technologies) was used to enrich total RNA preparation for small RNA molecules (< 200 bp). Total and small RNA were quantified using the ND-1000 spectrophotometer (Nanodrop, Wilmington, DE); RNA integrity and the content of microRNAs (%) in each sample were assessed by capillary electrophoresis utilizing the RNA 6000 Nano LabChip and the Small RNA Nano LabChip, respectively, using the Agilent Bioanalyzer 2100 (Agilent Technologies, Palo Alto, CA). Only total RNA samples with RNA Integrity Number (R.I.N.) values higher than 7 and the percentage of miRNA < 20% were used for microarray analysis.

### miRNA expression profiling

**Fig 1:**
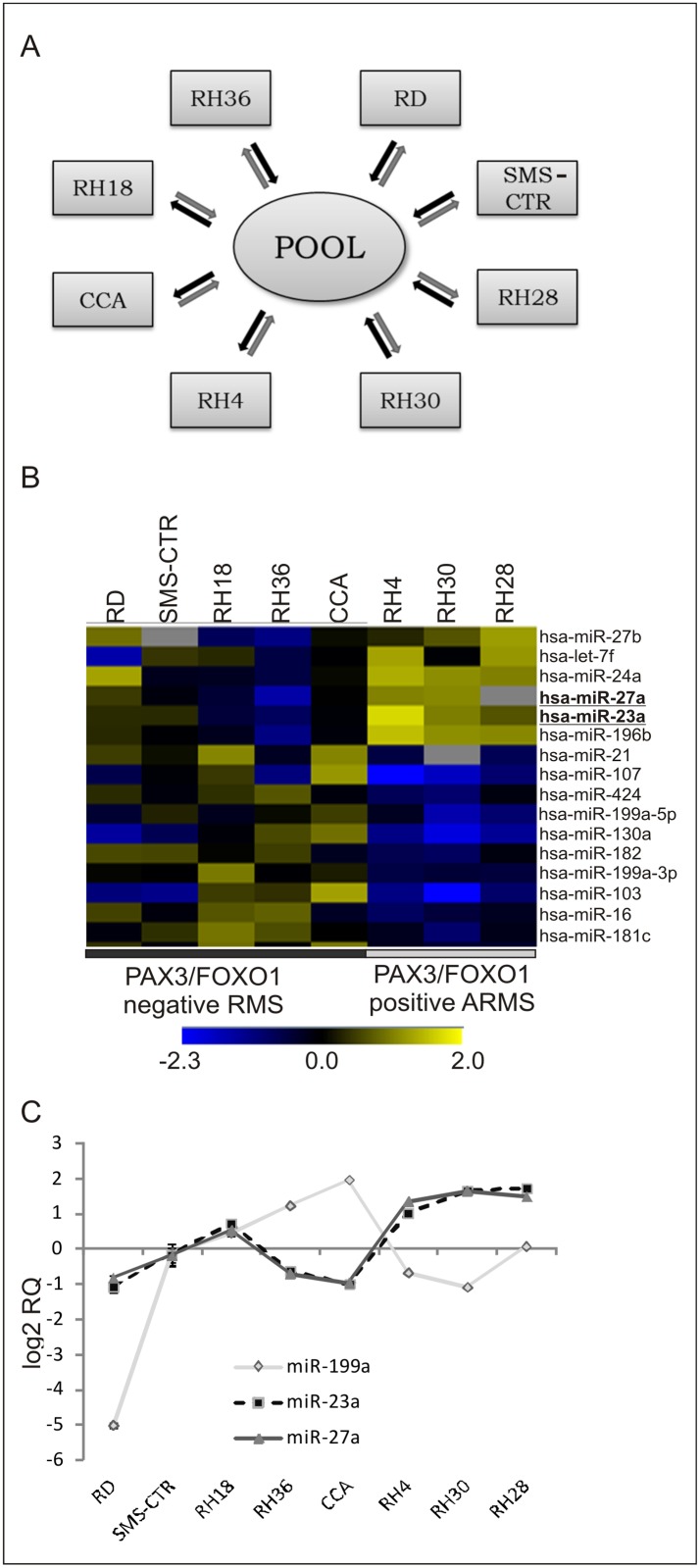
Differentially expressed miRNAs in RMS cell lines. **A**) Experimental design: the miRNA population from each cell line was compared to a common reference sample consisting of a balanced mixture of eight small RNA samples (< 200 nt) prepared from the same cell lines. Two replicates of each experiment were performed using different microarray slides in which sample and reference RNAs, labeled with Cy3 (gray arrow) or Cy5 (black arrow) fluorochromes, were crossed in both combinations (dye-swapping procedure). **B**) Heat map of 16 discriminant miRNAs between *PAX3/FOXO1* positive ARMS (in gray on the right, RH4, RH30, RH28) and negative (in dark grey on the left, RD, SMS-CTR, RH18, RH36, CCA) RMS cell lines identified by SAM analysis (*rows*: miRNAs; *columns*: RMS cell lines). A color-coded scale for the normalized expression values was used as follows: yellow and blue represent high and low expression levels in RMS cell lines with respect to a reference sample (a pool of eight RMS cell lines). The expression level of each miRNA was calculated as the ln (RMS cell line/Pool). **C**) Expression levels of miR-199a, miR-23a and miR-27a in eight RMS cell lines obtained with qRT-PCR. Two independent experiments were performed in triplicate. Results are shown as relative expression ratio obtained with the 2^-ΔΔCt^ method. RNU6B was used as reference miRNA. Vertical bars represent the 95% confidence interval (IC).

miRNA expression profiles were generated using our in-house array containing 386 probes for human, mouse, and rat miRNAs from the mirVana miRNA Probe Set (Ambion-Life Technologies). Negative control probes were designed using sequences from *Escherichia coli*; positive control probes (spikes) are sequences from *Mytilus galloprovincialis* (GEO Platform No. GPL17835). MiRNA expression profiles of eight different rhabdomyosarcoma cell lines were analyzed: four ARMS (RH4, RH28, RH30, RH18) and four ERMS (RD, RH36, CCA, SMS-CTR). The miRNA population from each cell line was compared with a reference sample consisting of a pool of the eight total RNA samples mixed in equal amounts. Two replicates of each experiment were performed using different microarray slides in which sample and reference RNAs, labeled either with Cy3 or Cy5 fluorochromes, were crossed in both combinations (dye-swapping procedure) (Fig 1A). Small RNA molecules (< 200 nt) were labelled with the mirVana Labeling Kit and amine-reactive dyes, as recommended by the manufacturer (Ambion-Life Technologies). Poly(A) polymerase and a mixture of unmodified and amine-modified nucleotides were used first to append a poly-nucleotide tail to the 3’ end of each miRNA. The amine-modified miRNAs were then cleaned up and coupled to NHS-ester modified Cy5 or Cy3 dyes (GE Healthcare, Little Chalfont, UK). Unincorpored dyes were removed with a second glass fibre-based clean-up procedure, as described by [42]. Microarray hybridization was carried out in a dual slide chamber (HybChamber, Gene Machines, San Carlos, CA, USA) humidified with 100 μL of 3×SSC. Labeled RNA was dissolved in 6 μl of 3X miRNA Hybridization Buffer (Ambion-Life Technologies), denatured at 95°C for 3 min. and applied directly to the slides. Microarrays were covered with a 24 × 24 mm cover slip and hybridized for 21 hours at 42°C by immersion in a high precision water bath (W28, Grant, Cambridge, UK). Hybridized slides were successively washed in: Low and High Stringency Buffer (Ambion-Life Technologies) for 30 sec. and dried by centrifugation (500 x g). Array scanning was carried out using a GSI Lumonics LITE dual confocal laser scanner with a ScanArray Microarray Analysis System (Perkin Elmer, Waltham, MA), and raw images were analyzed with QuantArray Analysis Software (GSI Lumonics, Ottawa, Canada). Raw data are available on the National Center for Biotechnology Information Gene Expression Omnibus (GEO) website (*http://www.ncbi.nlm.nih.gov/geo/*) using accession number GEO Series N. GSE52677.

### Gene expression profiling

Gene expression profiling was carried out in an ERMS cell line (RH36) transfected with pre-miR-27a or with a negative pre-control by using the ‘‘Whole Human Genome Oligo Microarray” (Agilent), consisting of 41,000 (60-mer) oligonucleotide probes, which span conserved exons across the transcripts of the targeted full-length genes. Each slide contains four individual microarrays, every one having about 44,000 features.

Eight hundred ng of total RNA were labeled with ‘‘Agilent One-Color Microarray-Based Gene Expression protocol” in accordance with the manufacturer’s instructions. Using 1.65μg of labeled cRNA to prepare the hybridization samples, the hybridization was carried out at 65°C for 17 hours in a hybridization oven rotator (Agilent). The arrays were washed with Agilent Gene expression wash buffers and Stabilization and Drying Solution, as suggest by the supplier. Slides were scanned on an Agilent microarray scanner (model G2565CA), and Agilent Feature Extraction software version 10.5.1.1 was used for image analysis. Raw data are available on the GEO website using accession number GSE52678.

### Statistical analysis of miRNA and gene expression data

Inter-array normalization of expression levels was performed with Variance Stabilizing Normalization (VSN) [43] for miR experiments and with quantile for gene expression profilings [44,45] in order to correct possible experimental distortions. The normalization function was applied to the expression data of all the experiments, and the values of spot replicates within the arrays were then averaged. With regard to gene expression data, Feature Extraction Software (Agilent) provides spot quality measures in order to evaluate the quality and the reliability of the hybridization data. In particular, flag "glsFound" (set to 1 if the spot had an intensity value that was significantly different from the local background or to 0 in any other case) was used to filter out unreliable probes: flag equal to 0 was to be noted as "not available (NA)." In order to make more robust and unbiased statistical analyses, probes with a high proportion of NA values were removed from the dataset. Seventeen percent of NA was used as the threshold in the filtering process, obtaining a total of 33,169 available human genes.

Cluster analysis and profile similarity searches were performed with Multi Experiment Viewer version 4.5.1 (tMev) of the TM4 Microarray Software Suite [46]. The level of each miRNA and transcript was calculated as ln (specific RMS cell line/Pool of RMS cell lines) and log2 (RH36 transfected cells/control), respectively. The identification of differentially expressed genes and miRNAs was performed using two class-Significance Analysis of Microarray (SAM) algorithm [47] with default settings. SAM uses a permutation-based multiple testing algorithm and identifies significant genes and miRNA with variable false discovery rates (FDR) which can be manually adjusted to include a reasonable number of candidate genes with acceptable and well-defined error probabilities. All heath maps were obtained by tMeV software using an unsupervised two-dimensional hierarchical clustering approach using average linkage with Pearson correlations. A color-coded scale for the normalized expression values was used as follows: yellow and blue represent high and low expression levels in RMS cell lines *vs*. a reference sample (pool of eight RMS cell lines), respectively. The expression level of each miRNA was calculated as the ln(RMS cell line/Pool).

### Target Prediction and Pathway Analysis

The TargetScan 5.1 [48] and mirSVR [49] algorithms were used to predict miR-27a targets. To identify the most likely targets, we focused our attention on putative mRNA differentially expressed in miR-27a over-expressing cells [19,50] and we performed a Gene Ontology (GO) analysis, using the DAVID tool [51], on the significant anti-correlated target genes identifying significantly enriched biological pathways (Modified Fisher Exact *p*-value < 0.05).

### qRT-PCR for miRNA or mRNA detection

Validation of differentially expressed miRNAs (microarray data) and miRNA detection for *in vitro* experiments was performed using the TaqMan miRNA Assay kit (Applied Biosystems-Life Technologies) [52]. Briefly, each RT reaction (15 μl) contained 10 ng of total purified RNA, 5X stem-loops RT primer, 1X RT buffer, 0.25 mM each of dNTPs, 50U MultiScribe reverse transcriptase and 3.8 U RNAse inhibitor. The reactions were incubated in a Mastercycler EP gradient S (Eppendorf, Hamburg, Germany) in 0.2 ml PCR tubes for 30 min at 16°C, 30 min at 42°C, followed by 5 min at 85°C, and then held at 4°C. The resulting cDNA was quantitatively amplified in 40 cycles on an ABI 7500 Real-Time PCR System, using TaqMan Universal PCR Master Mix and TaqMan MicroRNA Assays. Three replicates of each sample and endogenous control were amplified for each real-time PCR reaction.

For mRNA detection, 1 μg of total RNA was retrotranscribed with ImProm-II Reverse Transcription System (Promega) and qRT-PCRs were carried out using gene-specific primers and GoTaq qPCR Master Mix (Promega) using SYBR green chemistry by using a ABI 7500 Real-Time PCR System. RNU6B small nucleolar RNA or GADPH were chosen for miRNA or mRNA detection, respectively, as endogenous normalizers of the expression. The relative expression levels between samples were calculated using the comparative delta Ct (threshold cycle number) method (2^-ΔΔCt^) [53], implemented in the 7500 Real Time PCR System software.

### Flow Cytometric Analysis of the Cell Cycle

After transfection, miR-27a over-expressed or silenced cells and pre- or anti-control cells were harvested. For each sample, 1x10^6^ cells were fixed with 70% cold ethanol, washed in PBS, and incubated with propidium iodide (50 μg/mL) and RNase (100 μg/mL) for 60 minutes at 37°C. Samples were run in a BD FACScan (Becton Dickinson, Labware, Bedford, MA); the data were analyzed with ModFitLT V3.0 software (Verity Software House, Topsham, ME). Two independent samples were analyzed for each cell type.

### Migration and Invasion Transwell Assay

Migration was tested using cell culture inserts (Transwell) with an 8-μm pore size membrane (24-well format; Becton Dickinson). Chemoinvasion was measured using 24-well BioCoat Matrigel invasion chambers (Becton Dickinson) with an 8-μm pore polycarbonate filter coated with Matrigel. The lower compartment contained 0.5 ml of 1% serum medium conditioned by the NIH3T3 cell line as a chemoattractant or serum-free Dulbecco’s modified Eagle’s medium as a control. In the upper compartment, RH36 or RH4 cells per well were placed in triplicate wells and incubated for 18 hours at 37°C in a humidified incubator with a 5% CO2 atmosphere. After incubation, the cells on the upper surface of the filter were wiped off with a cotton swab; the cells on the lower surface were fixed in 2.5% glutaraldehyde, stained with 0.2% crystal violet in 20% methanol, and then photographed using a stereomicroscope (model MZ16; Leica Microsystems) equipped with a CCD camera. Images were elaborated with CorelDraw software (Corel, Ottawa, Canada), and the area occupied by the migrated cells was measured using ImageJ software (http://rsbweb.nih.gov/ij).

### Luciferase reporter assays

Luciferase reporter vectors containing the partial 3’-UTR of the indicated miR-27a target genes (*PHB*, *RBBP4*, *BCL7A*, *NKX3-1*, *HDAC9*, *RARA*, *RXRA*) were generated following PCR amplification of the 3’-UTR from human cDNA and cloned into the pmirGLO Dual-Luciferase miRNA Target Expression Vector (Promega). When indicated, the 3’-UTRs were mutagenized at the miR-27a recognition site/s using the QuickChange Multi Site- Directed Mutagenesis kit (Stratagene-Agilent Technologies, Palo Alto, CA) following the manufacturer’s instructions. MiR-27a-sensor was obtained by annealing, purifying and cloning short oligonucleotides containing three perfect miR-27a binding sites into the *Sac*I and *Xba*I sites of the pmirGLO vector.

About 8x10^4^ 293T cells were plated in 24-well plates and were co-transfected with 50 ng of the pmirGLO dual-luciferase (Promega) constructs containing the wild type or mutant/deleted 3’-UTRs of the indicated miR-27a potential target genes and 50 nM of pre-miR-27a or miRNA Precursor Molecules-Negative Control (all from Ambion-Life Technologies), using Lipofectamine2000 (Invitrogen-Life Technologies). Lysates were collected 48h after transfection and Firefly and Renilla Luciferase activities were measured with a Dual-Luciferase Reporter System (Promega). Relative luciferase activity was calculated by normalizing the ratio of Firefly/Renilla luciferase to negative control-transfected cells. Transfections were performed in triplicate and repeated 3–4 times.

## Results

### miRNAs expression profiling in RMS cell lines

We analyzed the miRNA expression profiles of eight different RMS cell lines: 3 *PAX3/FOXO1* positive ARMS (RH4, RH28, RH30), 1 *PAX3/FOXO1* negative ARMS (RH18) and 4 ERMS (RD, RH36, SMS-CTR, CCA) using the mirVana miRNA Probe Set V1 platform that is a collection of about 400 amino-modified DNA oligonucleotides. The miRNA population from each cell line was compared to the same reference sample consisting of an equal mixture of eight small RNA samples (< 200 nt) prepared from the same RMS cell lines (Fig 1A). We focused our attention on miRNA expression signature of *PAX3/FOXO1-*positive ARMS and translocation-negative RMS (GEO Series N. GSE52679). An unsupervised hierarchical clustering analysis showed specific miRNA expression signatures for *PAX3/FOXO1-*positive ARMS (RH4, RH28, RH30) and negative RMS (RD, CCA, SMS-CTR, RH36, RH18) cell lines. The SAM two-class analysis identified 16 miRNAs which discriminated between translocation-positive and negative RMS cell lines (Fig 1B). These results demonstrate that the miRNA platform was able to correctly classify different RMS subtypes and establishes the basis for the functional characterization of discriminating miRNAs. This result is in agreement with previous transcriptome studies that demonstrated that ERMS and ARMS have different expression signatures and that the presence of *PAX3/FOXO1* translocation confers a particular molecular signature to RMS samples [3,4,54]. Interestingly, a small set of miRNAs was able to successfully discriminate different RMS samples with respect to gene expression profiles such as demonstrated by Lu *et al*. in poorly differentiated solid tumors [55].

The expression levels of three miRNAs (miR-23a, miR-27a and miR-199a) which showed the greatest difference in expression between *PAX3/FOXO1* positive ARMS and negative RMS were validated by using TaqMan qRT-PCR and the Luminex xMAP technology, as described previously in [56]. The expression values were in agreement with microarray results (Fig 1C).

### miRNA expression levels in RMS tumor biopsies

Sarcoma microRNA Expression Database (S-MED) is a web-accessible database (http://www.oncomir.umn.edu/) that describes over 300 miRNA expression signatures in 22 different sarcoma types and corresponding normal tissue [30].

**Fig 2:**
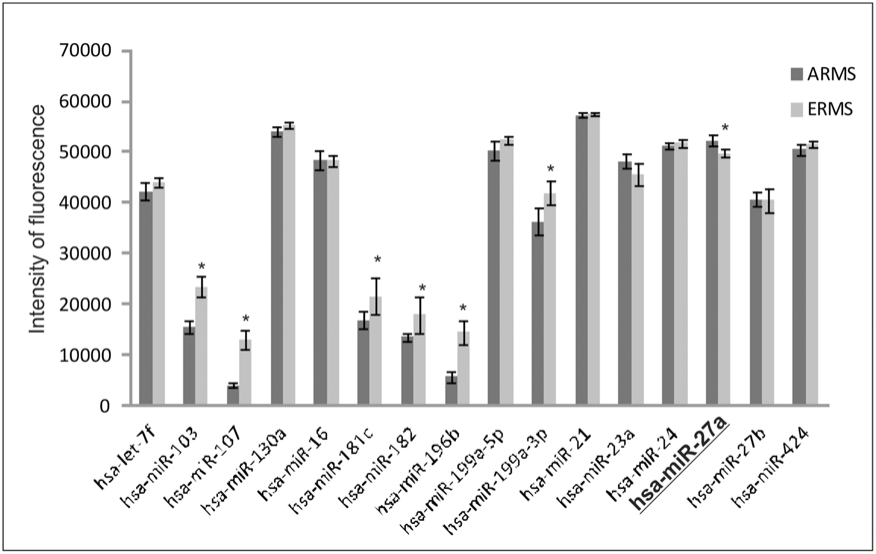
Expression levels of 16 miRNAs differentially expressed in RMS biopsies as deposited in S-MED Database. Expression data were quantile normalized across all S-MED data sets. T-test was performed to determine statistically significant differences in miRNA expression between ARMS patients (n = 19) and ERMS patients (n = 23) available in the S-MED Database. P-value were corrected using the Benjamini-Hochberg correction for multiple-testing (*FDR<0.1). MiR-27a is in bold and underlined.

The expression values of our 16 discriminating miRNAs in 42 RMS patients available in S-MED were downloaded. The data had already been quantile normalized across all S-MED data sets. A t-test was performed to identify differentially expressed miRNAs between translocation-positive ARMS and translocation-negative RMS. When a Benjamini-Hochberg correction for multiple-testing was applied, it was found that about 50% of discriminating miRNAs (miR-103, miR-107, miR-181c, miR-182, miR-196b, miR-199a-3p, miR-27a) identified in our signature (RMS cell lines) were confirmed in RMS patients (FDR<0.1), as can be seen in Fig 2. MiR-27a showed a significant up-regulation in translocation-positive ARMS *versus* ERMS patients suggesting that it plays a different role in the pathogenesis of translocation-positive and -negative RMS tumors. In order to elucidate the molecular function of miR-27a in this context, we performed a set of cellular and molecular experiments.

### miR-27a and miR-23a involvement in tumor cell growth, migration and invasion

**Fig 3:**
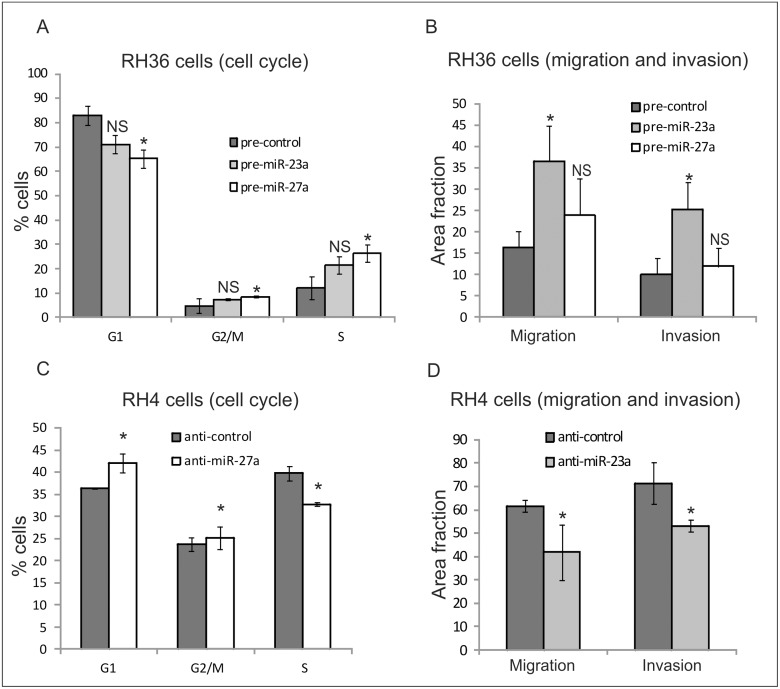
miR-27a and miR-23a involvement in tumor cell growth, migration and invasion. RH36 cells that express low levels of both miRNAs were transiently transfected with miR-27a or miR-23a precursors or their negative control (pre-control) and used to analyze proliferation (**A**) or migration and invasion (**B**). Conversely, RH4 cells, that over-express both miR-23a and miR-27a, were transiently transfected with inhibitors (anti-miR or anti- control) and used to analyze proliferation (**C**) or migration and invasion (**D**). The results are shown as mean±IC of the percentage (%) of proliferation, measured by flow cytometry atday 1 (A-C), and as the area covered by Transwell-migrating or Matrigel-invading cells (B-D). Cell proliferation, migration and invasion of miR-modulated cells are shown relative to control values. Two or three independent experiments were performed in triplicate and mean results are shown. *P<0.05; (IC, confidence interval); NS, not significant.

We transiently overexpressed pre-miR-27a in RH36, which is the ERMS cell line with the lowest endogenous expression level of this miRNA. Transient modulation was evaluated 24h post-transfection for expression and biological effects. Cell cycle progression of the transfected cells was assayed by flow cytometry. MiR-27a over-expressing cells showed an increase in the proliferation rate as well as a decrease in the percentage of cells in the G0/G1 phase (Fig 3A). Conversely, no significant effect was observed with regard to migration and invasion according to transwell assays (Fig 3B). We then decided to analyze the functional role of miR-23a since it was deregulated in our miRNA expression profiles and belongs to the same miRNA family of miR-27a. Interestingly, we observed that *in vitro* proliferation was not affected in the miR-23a over-expressing cells (Fig 3A), while the transwell assay showed an increase in cell movement compared to that in controls (Fig 3B). To confirm these results, we transiently induced the knockdown of miR-27a and miR-23a in RH4, which is the ARMS cell line with high endogenous expression levels of both miRNAs, and we evaluated cell cycle progression and cell motility respectively. We observed a decrease in the G0/G1 phase in cells treated with miR-27a inhibitors as well as a reduced motility of cells after transfection with miR-23a inhibitors with respect to controls (Fig 3C and 3D). Taken together, these results suggest that both miR-27a and miR-23a are involved in tumorigenic processes of RMS and contribute to promoting cell cycle progression and *in vitro* tumor cell invasion.

### Identification of miR-27a target genes

**Fig 4:**
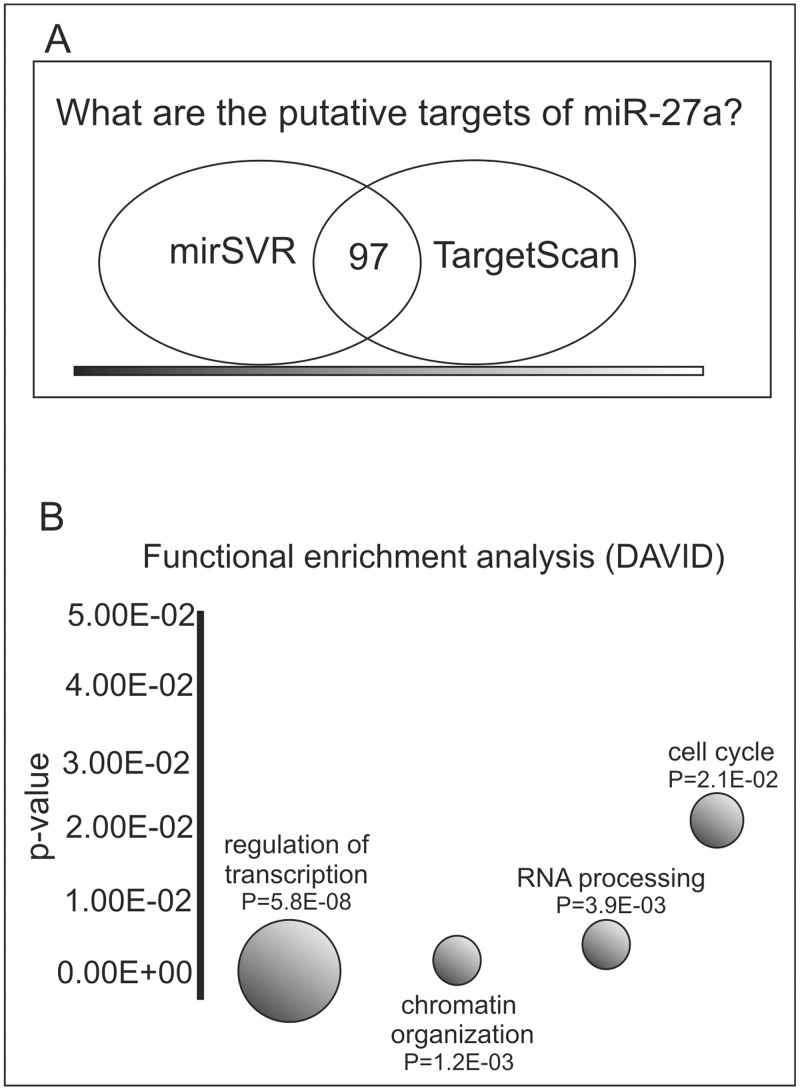
Identification of miR-27a putative target genes. **A)** miR-27a over-expressing RH36 cells were used to perform gene expression analysis by microarrays. SAM two-paired analysis identified 3,345 differentially expressed genes between miR-27a overexpressing cells with respect to controls. **A**) When differentially expressed genes with putative miR-27a targets obtained using the two prediction algorithms mirSVR and TargetScan 5.1 were crossed, a total of 507 genes, including 97 transcripts predicted by both algorithms, were found. **B**) Functional enrichment analysis was performed using the DAVID annotation tool, and the most enriched biological categories (modified Fisher exact *p-value* (EASE score) < 0.05) are shown. The area of each functional category (circle) is directly proportional to the number of genes. The complete list of 507 differentially expressed genes according to DAVID analysis is provided in S1 Table.

To identify genes directly or indirectly modulated by miR-27a, we defined the gene expression signatures of miR-27a over-expressing RH36 cells and pre-miR Negative Controls. SAM two-paired analysis identified 3,345 differentially expressed genes, uniformly distributed across up- and down-regulated, considering a 1.9% false discovery rate (FDR). A total of 507 genes, including 97 transcripts predicted by both algorithms, were found by crossing these results with putative miR-27a targets obtained from two target prediction algorithms such as mirSVR [49] and TargetScan 5.1 [57], (Fig 4A). The functional annotation web tool DAVID was used to analyze the 507 predicted targets in order to identify functional categories that are present in the expression signatures more frequently than expected by chance. A consistent number of genes involved in cell cycle (28 genes; P 2.1E^-2^), chromatin organization (20 genes, P 1.20E^-03^), RNA processing (24 genes, P 3.90E^-03^) and regulation of transcription (67 genes, P 4E^-5^) were identified (Fig 4B). A panel of 5 putative targets (*RARA*, *HDAC9*, *NKX3*-1, *RBBP4*, *PHB*), belonging to the above biological processes, predicted by at least one algorithm and present in one or more functional categories were selected for experimental validation. These putative target genes were also selected on the basis of their role in different aspects of the tumorigenic process, as has been described in the literature.

### miR-27a regulates the expression of some target genes

**Fig 5:**
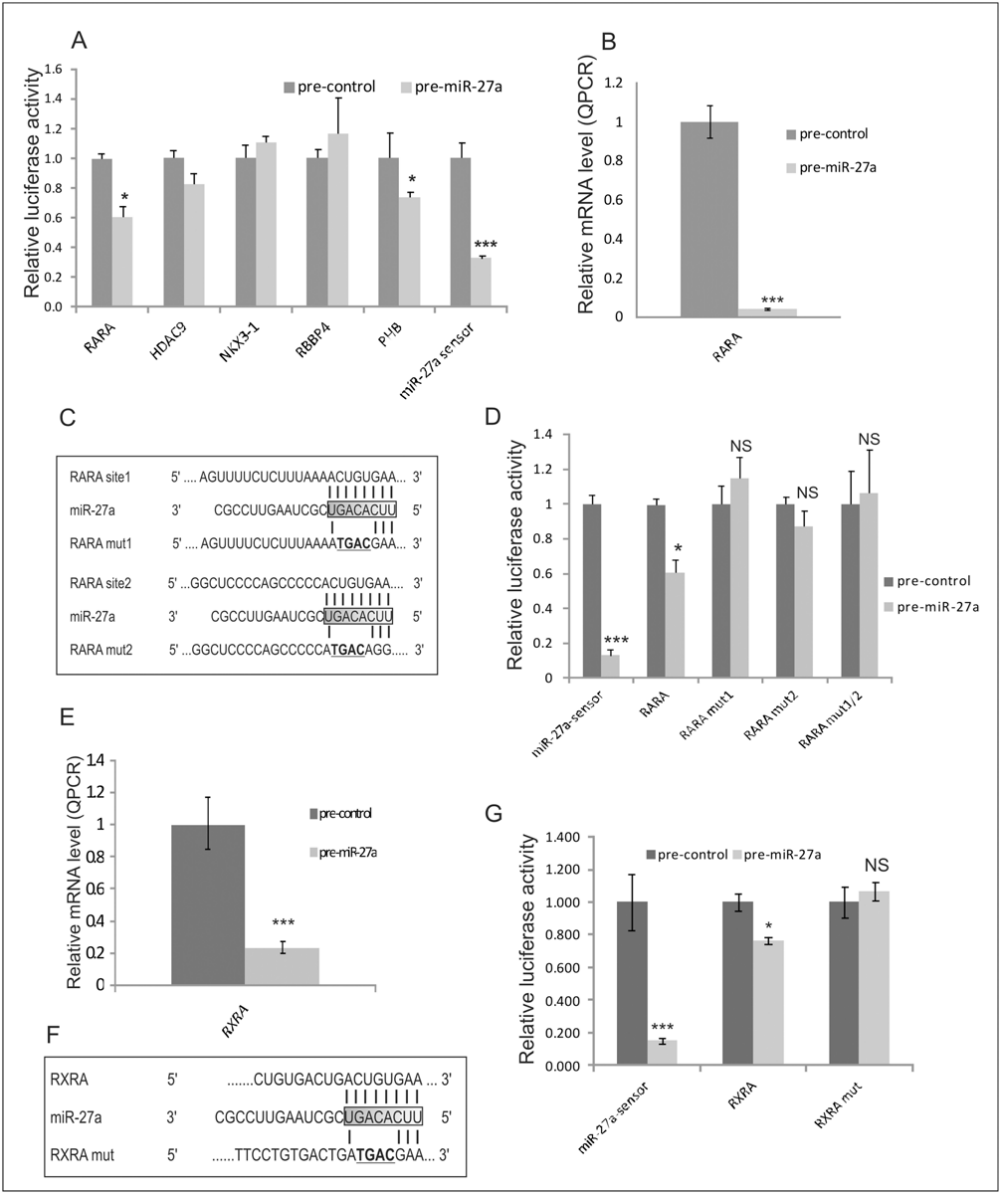
miR-27a modulates the expression of *RARA*, *RXRA* and *PHB* genes. **A)** 293T cells transfected with miR-27a precursors or negative controls (pre-miR-27a or pre-control) analyzed 48h post-transfection were used to perform Luciferase assays in cells transfected with reporter constructs containing the wild type for the indicated genes or a synthetic sequence including a perfect miR-27a binding site (sensor). Results from three independent experiments are shown as mean ± IC of Firefly luciferase activity relative to controls, normalized on Renilla luciferase activity. *P<0.05; **P<0.01; ***P<0.001. **B**) *RARA* mRNA levels measured by qRT–PCR in miR-27a over-expressing RH36 cells (pre-miR-27a, light gray box) and negative controls (pre-control, dark gray box) analyzed 24h post-transfection. **C**) Reporter constructs containing miR-27a wild type or mutant binding sites RARA 3’-UTRs (RARA mut1 and RARA mut2). **D**) 293T cells transfected with miR-27a precursors (pre-miR-27a or pre-control) analyzed 48h post-transfection were used to perform Luciferase assays in cells transfected with reporter constructs containing miR-27a wild type (RARA) or mutant binding sites in RARA 3’-UTRs (RARA mut1, RARA mut2 and RARA mut1/2). Results from three independent experiments are shown as mean ± IC of Firefly luciferase activity relative to controls, normalized on Renilla luciferase activity. NS = not significant; *P<0.05; **P<0.01; ***P<0.001 (IC = confidence interval). **E**) *RXRA* mRNA levels measured by qRT–PCR in miR-27a over-expressing RH36 cells (pre-miR-27a, light gray box) and negative controls (pre-control, dark gray box) analyzed 24h post-transfection. **F**) Reporter constructs containing miR-27a wild type (RXRA) or mutant binding sites in RXRA 3’-UTRs (RXRA mut). **G**) 293T cells transfected with miR-27a precursors (pre-miR-27a or pre-control) analyzed 48h post-transfection were used to perform Luciferase assays in cells transfected with reporter constructs containing miR-27a wild type (RXRA) or mutant binding sites in RXRA 3’-UTRs (RXRA mut). Results from three independent experiments are shown as mean ± IC of Firefly luciferase activity relative to controls, normalized on Renilla luciferase activity. NS, not significant; *P<0.05; **P<0.01; ***P<0.001

Direct modulation by miR-27a of its targets was confirmed by performing luciferase assays in miR-27a-overexpressing or control 293T cells transfected with reporter vectors containing wild type or mutated 3’-UTRs. A miR-27a-sensor construct was used as a positive control. We observed a significant reduction in luciferase activity in constructs containing the prohibitin (*PHB*), and retinoic acid receptor alpha (*RARA*) 3’-UTRs. Luciferase expression driven by the 3’-UTRs of retinoblastoma binding protein 4 (*RBBP4*), histone deacetylase 9 (HDAC9), B-cell CLL/lymphoma 7A (*BCL7A*) and NK3 homeobox 1 (*NKX3-1*) were instead not significantly repressed demonstrating that miR-27a is not directly involved in the modulation of these putative targets (Fig 5A). Interestingly, PHB has already been demonstrated to be a direct target of miR-27a, as has been noted in the miRecords database [58]. Furthermore, we found a very strong reduction of *RARA* expression levels in miR-27a overexpressing 293T cells 24h post-transfection by means of qRT-PCR technology (Fig 5B). As detected by TargetScan 5.1 analysis, RARA 3’-UTR is characterized by two different binding sites for miR-27a. We prepared a series of luciferase constructs in which one or both miR-27a binding sites were mutated in four contiguous positions in order to demonstrate the real interaction between miR-27a and *RARA* (Fig 5C). The luciferase reporter assays in 293T cells demonstrated that the mutated binding sites abrogated the negative effect of overexpressed miR-27a on RARA 3’-UTR (Fig 5D), indicating a specific, direct regulation of miR-27a at both RARA 3’-UTR binding sites. RARA acts as heterodimers with the retinoic X receptor family. Although we did not identify *RXR*s among the deregulated transcripts in microarray experiments from miR-27a overexpressing cells, RXRA was indicated as a potential target gene of miR-27a by TargetScan analysis. We thus decided to experimentally test if RXRA is a target of miR-27a in the rhabdomyosarcoma cell line and found a marked decrease in *RXRA* expression levels in miR-27a overexpressing 293T cells, 24h post-transfection, when measured by qRT-PCR (Fig 5E). Luciferase assays also revealed a 20% reduction in luciferase expression driven by the 3’-UTRs of *RXRA*, an effect that was abrogated by point mutations in the miR-27a interacting region of the 3’-UTR of RXRA (Fig 5F and 5G).

## Discussion

A number of studies have investigated the different behavior and molecular traits of ARMS and ERMS. These two types of tumors showed different regulation of cell cycle [59,60] and distinct expression of oncogenes, including IGFBP2, ALK, MYCN and plakoglobin [61,62,63,64]. Our previous studies on the human RMS transcriptome have, in fact, indicated that the *PAX/FOXO1* fusion gene gives a particular expression signature to ARMS [3,54]. Emerging data have, moreover, indicated that miRNA expression patterns could be useful in improving the classification of cancers and in predicting their outcome. Gasparini *et al*, for example, identified a 4-miRNA signature, which included miR-27a, able to stratify triple negative breast cancers into high and low risk groups and thus confirmed the immense potential of miRNAs in opening new avenues to detect and diagnose early-stage cancers [65].

Although there are studies that have shown that some miRNAs acting as key regulators of skeletal muscle cell fate determination are de-regulated in both alveolar and embryonal RMS [20,22], the role of miRNAs in RMS tumorigenesis remains poorly understood. The study outlined here set out to analyze miRNAs expression profiles in human rhabdomyosarcoma cell lines in which a set of 16 miRNAs able to discriminate translocation-positive ARMS from translocation-negative RMS were identified. MiRNA expression microarray data from tumor biopsies deposited in the S-MED, a Sarcoma microRNA Expression Database that collects expression profiles for over 700 miRNAs in various human sarcoma types measured by Illumina bead arrays [66] were evaluated. Out of the 50 sarcoma subtypes contained in S-MED, we used the microarray data of 42 RMS tissue samples to evaluate the differences in expression patterns between translocation-positive ARMS and translocation-negative RMS. Interestingly, it was found that about 50% of discriminating miRNAs in RMS cells obtained in these experiments were also confirmed in RMS patients (FDR<0.1), and miR-27a was one of the most over-expressed in translocation-positive ARMS with respect to translocation-negative RMS.

Our functional studies on miR-27a over-expressing cells uncovered a significantly enhanced proliferation rate that was paralleled by a decrease in the percentage of cells in the G1 phase. In agreement with our findings, recent studies have supported the oncogenic role of miR-27a. MiR-27a has, for example, exhibited oncogenic activity in breast cancer by down-regulating zinc finger ZBTB10 protein leading to over-expression of pro-survival and pro-angiogenic genes including *survivin*, *VEGF*, *VEGF receptor 1* (*VEGFR1*) [36]. High levels of miR-27a have also been observed in MCF-7 breast cancer cells in which the level of FOXO1 protein is very low, demonstrating a novel mechanism of FOXO1 regulation mediated by miR-27a that may contribute to the oncogenic transformation or maintenance of an oncogenic state in breast cancer [67]. Some have hypothesized that the overexpression of miR-27a affects cell cycle progression by interacting with the tumor suppressor FBW7 that regulates ubiquitylation and turnover of the FBW7 target cyclin E [68]. Inhibition of miR-27a was, moreover, found to suppress the growth, colony formation, and migration of pancreatic cancer cells by targeting Spry2 [69]. MiR-27a has been identified in adenocarcinoma as an oncogene that increases cell growth by directly targeting tumor suppressor prohibitin (PHB) [34]. Prohibitin physically interacts with all Rb family proteins *in vitro* and *in vivo* and has been found to be very effective in repressing E2F-mediated transcription resulting in cell growth arrest [70]. MiR-27a has also been found to be an androgen-regulated oncomir in prostate cancer that acts *via* PHB to increase cell growth [71].

In the study outlined here we first performed computational analyses using mirSVR and TargetScan algorithms to determine the miR-27a target genes. Since a large fraction of false positives were generated, we decided to integrate target predictions with gene expression profiles of miR-27a over-expressing cells. Already known targets, such as PBH that seems to have a tumor suppressor role in RMS as well as in other tumors, were confirmed. At the same time, retinoic acid receptor alpha (RARA) and retinoic X receptor alpha (RXRA) have been demonstrated, for the first time in RMS, to be real miR-27a targets.

Retinoic acid receptors (RARα, β and γ) are nuclear hormone receptors that interact as heterodimers with retinoic X receptors (RXRs). RXR-RARA heterodimers act as ligand-dependent transcriptional regulators by binding to the specific retinoic acid response element (RARE) found in the promoter of target genes. Retinoid acids (RA), also known as ATRA, interact with retinoic acid receptors (RAR) inducing growth inhibition together with differentiation in many cell types and are seen as promising candidate agents for the prevention and treatment of several human cancers. They have, in fact, already been used in clinical trials evaluating, in particular, the treatment of acute promyelocytic leukemia and the prevention of head and neck, cervical, and lung cancers [24,72,73,74]

RMS cells fail to complete the skeletal muscle differentiation program and to irreversibly exit the cell cycle. Indeed, understanding how retinoid acids affect these pathways may yield insights into both physiological and pathophysiological processes of this tumor. Several *in vitro* studies have demonstrated that retinoic acid influences cell proliferation and muscle gene expression in human RMS cell lines [29,75]. ATRA treatment of human RMS xenografts in immuno-suppressed NOD-SCID (NSG) mice, instead, has been found to be associated to enhanced muscle cell differentiation but not complete cell cycle arrest [76]. These findings suggest that retinoic acid alone is unlikely to be beneficial as a single-agent therapy in inducing RMS differentiation but may improve survival when combined with standard cytotoxic therapy. It has also been demonstrated that the effectiveness of RA cancer treatment depends on the expression of RA receptors [23]. A correlation between RA-receptor expression and RA sensitivity may explain retinoids’ effective action in rhabdomyosarcoma.

Another important finding concerns the use of retinoids to treat the metastatic progression of rhabdomyosarcoma. An *in vivo* study demonstrated that ATRA treatment, administered prior to and following primary tumor excision, was associated with a reduction in the metastatic potential of rat S4MH rhabdomyosarcoma. This, of course, suggests that a therapeutic approach based on pre- and post-surgery of retinoid therapy could contribute to controlling both recurrence and metastatic progression [77]. These lines of research confirm that miR-27a may be a potential therapeutic target in RMS. In fact, silencing miR-27a to decrease its abnormal over-expression in RMS cell lines could restore normal retinoic acid receptor expression and improve the response to retinoic acid treatment. This hypthesis has been supported by other studies designed to analyze the involvement of miR-27a in multidrug resistance and response to chemotherapy in tumor cells. MiR-27a appears, in fact, to be involved in the activation of P-glycoprotein, the MDR1 gene product that confers cancer cell resistance to a broad range of chemotherapeutics in ovarian and esophageal cancer [78,79]. As patients with high miR-27a expression levels have also shown a poor response to first-line chemotherapy in metastatic and recurrent gastric cancer, miR-27a could be used as a biomarker to predict the response to chemotherapy and subsequent prognosis in patients with gastric cancer [37]. Chintharlapalli and colleagues demonstrated, for the first time, that CDODA-Me, a synthetic derivative of glycyrrhetinic acid, acts by downregulating miR-27a that is accompanied by an enhanced expression of ZBTB10 and Myt-1 which produces cell cycle arrest in colon cancer cells. Some studies have outlined the potential clinical applications of CDODA-Me-miR interactions for cancer treatment [80]. Taken together, these studies have demonstrated that pharmacological approaches to target miR-27a might offer novel therapeutic opportunities for the treatment of multidrug resistance cancers.

In conclusion, our results demonstrated that 16 miRNAs were able to correctly classify tumors into tumor subtypes (*PAX3/FOXO1-*positive ARMS and translocation-negative RMS) and may be useful to diagnose RMS. MiR-27a showed the highest expression level and worse prognosis in patients with translocation-positive ARMS cell lines. Since retinoic acid receptor alpha and retinoid X receptor alpha were found to be novel targets of miR-27a, suggesting a potential role of miR-27a in therapy of rhabdomyosarcoma.

## Supporting Information

S1 Table List of miR-27a predicted differentially expressed genes that were included by DAVID analysis in statistically significant functional categories. (XLSX)
